# Thaliporphine Preserves Cardiac Function of Endotoxemic Rabbits by Both Directly and Indirectly Attenuating NFκB Signaling Pathway

**DOI:** 10.1371/journal.pone.0039174

**Published:** 2012-06-25

**Authors:** A. S. Lee, W. P. Chen, Y. L. Kuo, Y. J. Ho, S. S. Lee, M. J. Su

**Affiliations:** 1 Division of Cardiology, China Medical University Hospital, No.2, Taichung, Taiwan; 2 L5 Research Center, China Medical University Hospital, No.2, Taichung, Taiwan; 3 China Medical University, No.91, Taichung, Taiwan; 4 Institute of Pharmacology, College of Medicine, National Taiwan University, No.1, Sec.1, Taipei, Taiwan; 5 School of Pharmacy, College of Medicine, National Taiwan University, No.1, Sec.1, Taipei, Taiwan; University of Western Ontario, Canada

## Abstract

Cardiac depression in sepsis is associated with the increased morbidity and mortality. Although myofilaments damage, autonomic dysfunction, and apoptosis play roles in sepsis-induced myocardial dysfunction, the underlying mechanism is not clear. All of these possible factors are related to NFκB signaling, which plays the main role in sepsis signaling. Thaliporphine was determined to possess anti-inflammatory and cardioprotective activity by suppressing NFκB signaling in rodents. The purpose of this study is to further prove this protective effect in larger septic animals, and try to find the underlying mechanisms. The systolic and diastolic functions were evaluated *in vivo* by pressure-volume analysis at different preloads. Both preload-dependent and -independent hemodynamic parameters were performed. Inflammatory factors of whole blood and serum samples were analyzed. Several sepsis-related signaling pathways were also determined at protein level. Changes detected by conductance catheter showed Thaliporphine could recover impaired left ventricular systolic function after 4 hours LPS injection. It could also reverse the LPS induced steeper EDPVR and gentler ESPVR, thus improve Ees, Ea, and PRSW. Thaliporphine may exert this protective effect by decreasing TNFα and caspase3 dependent cell apoptosis, which was consistent with the decreased serum cTnI and LDH concentration. Thaliporphine could protect sepsis-associated myocardial dysfunction in both preload-dependent and -independent ways. It may exert these protective effects by both increase of “good”-PI3K/Akt/mTOR and decrease of “bad”-p38/NFκB pathways, which followed by diminishing TNFα and caspase3 dependent cell apoptosis.

## Introduction

Septic shock is an increasingly severe clinical syndrome and the primary cause of death in intensive care units characterized by hemodynamic dysfunction of several organs [1], [2]. This is due to more and more uses of diagnostic or therapeutic invasive procedures and the application of immunosuppressive chemotherapy. It develops from initial normal host response to the infection which is amplified and dysregulated. The features of sepsis include fever, diminished urine output, thrombocytopenia, hypotension, and multiple organ failure including myocardial dysfunction [3]. Severe septic shock presents with impaired systolic function, abnormal diastolic function of heart, marked peripheral vasodilatation, and decrease in central blood volume [4], [5]. The pathogenesis of the myocardial dysfunction in sepsis is multifactorial, both intrinsic myocardial depression [6] and factors secondary to the acidosis, hypotension, hypoxia and circulating inflammatory factors (IL-1β, TNFα, platelet-activating factor, lysozyme, nitric oxide, and free radicals) may play important roles [7], [8], [9]. Lipopolysaccharide (LPS), a major component of the outer membrane of Gram negative bacteria, activates several intracellular signaling pathways including mainly NFκB and MAP kinase signaling, leading cells to a pro-inflammatory state with the secretion of cytokines and overexpression of several markers of the immune response [10]. The release of different cytokines may be responsible for both intrinsic and extrinsic apoptosis pathways. Recently, some evidences suggest that LPS also involved in the PI3K pathway followed by mammalian target of rapamycin (mTOR) signaling [11], which could negatively regulate p38 dependent matrix metalloproteinase-9 (MMP9) activation.

Thaliporphine, a phenolic aporphine alkaloid isolated from plants of several families such as *Lauracea* [12], possesses protective effects on multiple organs of LPS-induced endotoxemic rodents in our previous study [13]. It could suppress NO, superoxide anion, and TNFα production, and decrease infiltration of inflammatory cells into injured tissues. In our recent study, we also found Thaliporphine could ameliorate cardiac depression through attenuating toll-like receptor 4 (TLR4) signaling and decreasing downstream NFκB activation. This effect of Thaliporphine further prevented cardiac sarcoplasmic reticulum Ca^2+^-ATPase (SERCA2) function from nitrosylation by peroxynitrite via decreasing iNOS and TNFα expression of endotoxemic rats [14]. However, the detailed underlying mechanism of Thaliporphine on endotoxemic hearts remains to be further examined, especially in larger animals such as rabbits and dogs. For the present study, we used LPS-induced endotoxemic rabbits, an animal model recognized to mimic abnormal cardiac function of human sepsis, to investigate the acute protective effects of Thaliporphine and the underlying mechanisms.

## Materials and Methods

### 1. Thaliporphine Synthesis

Thaliporphine was synthesized from glaucine and purified by column chromatography, followed by identification with spectrometric methods (mass, NMR). Its purity (>99%) was established by HPLC with retention time of 15.17 min under the following conditions: Lichrospher RP-18e, 5 µm, 4.0×125 mm; eluent MeOH-0.01 M KH_2_PO_4_/H_2_O (pH 7.02) 60∶40; flow rate 1.0 ml/min; injection volume 20 µl (1 mg/ml); detection 218 nm.

### 2. Animals

This study was approved by National Taiwan University IACUC and performed in accordance with the *Guide for the Care and Use of Laboratory Animals* published by the US National Institutes of Health (NIH Publication No. 85-23, revised 1996). Male New Zealand White rabbits weighing 1.2 to 1.5 kg were housed in individual cages in a controlled environment with free access to food and water. The rabbits were randomly divided into 5 groups: Sham, LPS 1 mg/kg (LPS), LPS pretreated with 1 mg/kg Thaliporphine (LPS/T1), LPS pretreated with 3 mg/kg Thaliporphine (LPS/T3), 3 mg/kg Thaliporphine alone (T3). There are six animals in each group. Endotoxemia was induced by intravenous injection of lipopolysaccharides from *Escherichia coli, Salmonella enteritidis, and Salmonella minnesota* (Sigma Chemical, St. Louis, MO) 1 mg/kg through marginal ear vein of rabbits which were fasted from the night before the experiment began. The cardiac function and blood analysis were monitored 4 hours after LPS injection with or without 30 min pretreatment of 1 or 3 mg/kg Thaliporphine.

### 3. Analysis of Hemodynamic Characteristics in vivo

All groups of animals were anesthetized with 3% isoflurane under mechanical ventilation. After tracheostomy, a micro tip pressure-volume catheter (3.0F; Scisense Instrument, ON, Canada) was inserted into the right carotid artery to measure blood pressure and flow under stable condition for 5 min, and then advanced into the left ventricle under pressure monitor. Signals of pressure and volume associated with lead II EKG were continuously recorded at the sampling rate of 1000 Hz using PONEMAH real-time acquisition interface P3P Plus coupled to a digital converter (ACQ-16). All pressure-volume loop data were analyzed by using a program of PONEMAH Life Sciences Suite from Data Sciences International (DSI Corporate Headquarters, St. Paul, MN). The hemodynamic parameters included heart rate (HR), mean arterial pressure (MAP), left ventricular end-systolic pressure (LVSP), maximal velocity of pressure rise (+dP/dt), and maximal velocity of pressure fall (−dP/dt). The contractility parameters were determined under conditions of intermittent changing preload, elicited by transiently inflating a balloon previously inserted into abdominal inferior vena cava through femoral vein. These data allowed computer calculation of preload-independent variables such as time-varying end-systolic elastance (Ees), arterial elastance (Ea), and preload-recruitable stroke work (PRSW).

### 4. Measurement of Blood and Serum Parameters

Blood samples were collected from carotid artery at the end of experiments and then centrifuged at 10000 rpm for 5 min to obtain serum. Blood pH and cardiac troponin I was measured by *i*STAT Portable Clinical Analyzer with G3+ and cTnI cartridge (Abbott Point of Care Inc. Princeton, NJ, USA). Blood glucose was measured by strips of OneTouch blood glucose meter (LifeScan Ltd., Canada). Serum LDH (CytoTox 96, Non-Radioactive Cytotoxicity Assay, Promega Corporation, WI, USA) was measured according to the manufacturer’s instructions. WBC count was measured on a hematology analyzer in whole blood mode (KX-21N, Sysmex, MA, USA). The number of PMNs was calculated by multiplying the percent PMN in the sample by the total WBC count.

**Table 1:** Effects of thaliporphine on basic and blood parameters of endotoxemic rabbits.

Group	Sham	LPS	LPS/T1	LPS/T3	T3
Body Weight (kg)	1.38±0.04	1.37±0.05	1.34±0.05	1.33±0.06	1.28±0.03
Blood pH	7.51±0.02	7.45±0.01*	7.46±0.04	7.55±0.02^##^	7.50±0.01
Blood Glucose (mg/dL)	157.00±12.73	68.17±13.54**	114.67±13.29^#^	143.17±2.95^##^	149.75±8.01
cTnI (ng/mL)	1.08±0.45	8.70±1.76**	6.44±1.74	3.98±0.86^#^	1.40±0.12
LDH (O.D.)	1.31±0.06	2.20±0.18**	2.22±0.21	1.75±0.12^#^	1.40±0.10
WBC (10^9^/L)	6.6±1.57	3.86±0.45*	5.02±1.20	4.36±1.06	5.90±0.25
PMN (%)	24.12±1.71	32.78±4.23**	23.18±4.43	26.93±0.85	23.28±1.86
PMN Count (10^9^/L)	1.59±0.10	1.28±0.01*	1.10±0.23	1.36±0.22	1.37±0.09

Values are means±S.E. cTnI, cardiac troponin I; LDH, lactate dehydrogenase; WBC, white blood cell; PMN, polymorphonuclear neutrophil. * *P*<0.05 and ** *P*<0.02 as compared with sham group; ^#^ *P*<0.05 and ^##^ *P*<0.02 as compared with LPS group.

### 5. Total Protein Extraction of Myocardial Tissues and Western Blotting

Ventricles from all groups of rabbits were homogenized by using a Dounce homogenizer in RIPA lysis buffer (137 mM NaCl, 5 mM EGTA, 5 mM EDTA, 25 mM Tris, 0.5% sodium deoxycholate, 0.1% SDS, and 0.1% Triton X-100, adjusted to pH7.5), containing protease inhibitor cocktail for mammalian cell and tissue extracts (Sigma-Aldrich Co., USA). The lysates were disrupted in advance by sonication at 4°C for 30 min, and centrifuged at 14000 rpm for 10 min at 4°C to collect the supernatant as total protein extract. Protein concentrations were determined by using Bradford Protein Assay kit (Bio-rad Laboratories Inc, CA, USA).

Samples of equal protein content (100 µg each lane) were subjected to SDS-PAGE and electrotransferred to PVDF membranes (Immobilon-P membranes, Millipore Corp., Bedford, MA, USA). The primary antibodies employed included MMP9, mTOR, p38, phospho-p38 (Thr 180/Tyr 182), PI3K, Akt, phospho-Akt (Ser 473), α-tubulin, Bcl2, phospho-NFκB p65 (Ser 536), TNFα (Santa Cruz Biotechnology, Inc. Santa Cruz, CA, USA), and β-actin (Sigma-Aldrich Co., USA). The detection of specific proteins in immunoreactive bands was carried out by enhanced chemiluminescence (ECL kit, PerkinElmer Life Sciences, Inc, Boston, MA) and quantified by GelDoc-It imaging system (UVP, CA, USA).

### 6. Nuclear Protein Extraction and Electrophoretic Mobility Shift Assay (EMSA)

Nuclear protein extracts were obtained by using Nuclear Extraction Kit (Panomics Inc., Dumbarton Circle Fremont, CA) according to the manufacturer’s instruction with its supplemented protease and phosphatase inhibitors. LightShift® Chemiluminescent EMSA Kit was used to identify the interaction of NFκB with DNA. Nuclear proteins (10 µg) were incubated with the biotin-labeled probe (AGTTGAGGGGACTTTCCCAGGC) and subjected to 6% nondenaturing polyacrylamide gel electrophoresis. After transferring to nylon membrane, the oligos on the membrane were fixed using UV cross-linker. The blot was then blocked and incubated in streptavidin–HRP containing mixture.

### 7. TUNEL Staining for Apoptosis

Hearts from all groups of rabbits were removed and immersed in 4% paraformaldehyde for overnight. The hearts were then embedded in molten paraffin, and serial 5 µm myocardial sections were cut. The sections were placed on poly-L-lysine-coated slides (4 sections per slide). The paraffin-embedded sections were dewaxed by immersion in xylene for 10 min and were sequentially rehydrated by graded ethanol. The antigen was retrieved with proteinase K method. Terminal deoxynucleotidyl transferase dUTP nick end labeling (TUNEL) staining was performed using a commercial kit (In Situ Cell Death Detection Kit, Fluorescein, Roche Diagnostics.) with modification of the recommended protocol. The fluorescein-12dUTPlabeled DNA was directly visualized and analyzed by fluorescence microscopy (Olympus IX70 research inverted system microscope).

### 8. Caspase 3 Activity of Myocardial Tissues

Caspase 3 Colorimetric Activity Assay Kit, DEVD (Chemicon International, Inc, USA) was used to measure caspase 3 activity by microplate ELISA reader (VICTOR X, PerkinElmer, Waltham, MA, USA) according to the manufacturer’s instructions.

**Figure 1:**
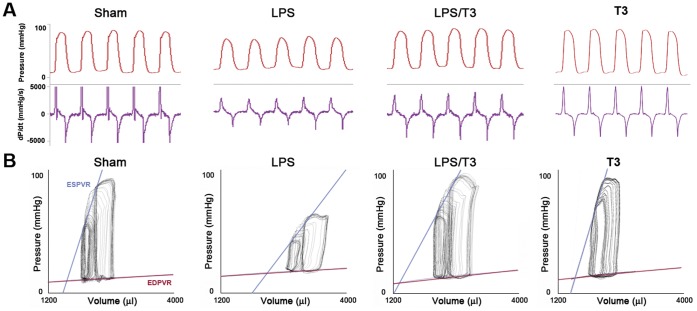
Thaliporphine could significantly ameliorate depressed hemodynamic characteristics of endotoxemic rabbits. **A**. Pressure (top) and dP/dt (bottom) signals obtained with the pressure-volume conductance catheter system from sham, LPS (1 mg/kg), LPS plus 3 mg/kg Thaliporphine (LPS/T3), and 3 mg/kg Thaliporphine alone (T3) treated rabbits. **B**. Representative P-V loops at different preloads, showing differences in both end-systolic P-V relation (ESPVR) and end-diastolic P-V relation (EDPVR) between three groups. The less steep ESPVR and increased EDPVR in LPS treated animals indicate decreased contractile function and increased diastolic stiffness. Co-administration of 3 mg/kg Thaliporphine could reverse both cardiac depressive conditions.

**Table 2:** Effects of thaliporphine on hemodynamic parameters of endotoxemic rabbits.

Group	Sham	LPS	LPS/T1	LPS/T3	T3
HR (bpm)	285.50±14.37	312.67±15.03	302.83±13.60	310.50±5.86	290.25±17.21
MAP (mmHg)	78.61±1.68	64.48±4.69*	60.67±2.20*	69.93±4.95*	80.94±1.56*
LVSP (mmHg)	96.60±4.92	75.08±4.04**	75.32±2.17**	89.95±2.44^##^	103.7±7.20
+dP/dt (mmHg/s)	5276.80±777.21	2988.08±251.31**	2762.48±312.45**	4075.10±374.31^#^	5720.18±721.28
−dP/dt (mmHg/s)	6413.75±629.40	3442.97±514.73**	3525.88±554.76**	4720.70±400.39^#^	6428.75±734.02
Ees (mmHg/mL)	85.6±7.8	49.9±4.8**	57.8±8.5**	69.5±5.3^#^	81.1±3.1
Ea (mmHg/mL)	277.0±29.7	165.5±30.2**	227.7±24.3	238.1±21.6^##^	281.6±10.3
PRSW (mmHg)	26.97±7.75	14.13±2.56**	20.90±4.22	27.23±3.81^##^	29.12±6.64

Values are means±S.E. HR, heart rate; MAP, mean arterial pressure; LVSP, left ventricular systolic pressure; +dP/dt, maximal velocity of pressure rise; −dP/dt, maximal velocity of pressure fall; Ees, end systolic elastance; Ea, arterial elastance; PRSW, slope of preload recruitable stroke work. * *P*<0.05 and ** *P*<0.02 as compared with sham group; ^#^ *P*<0.05 and ^##^ *P*<0.02 as compared with LPS group.

### 9. Cytokine Protein Array

To determine levels of select cytokines and chemokines, cytokine array membranes (R&D Systems, Minneapolis, MN) were treated with serum from each group. Membranes were then developed with enhanced chemiluminescence (ECL) reagents (Millipore, Billerica, MA), and images were acquired and analyzed with the G-box imaging system (Syngene, India).

### 10. Statistical Analysis

The results were presented as means ± standard error of the mean (S. E.M.). Significant differences between groups were determined by one-way ANOVA with post hoc analysis using the Bonferroni *t*-test.

**Figure 2:**
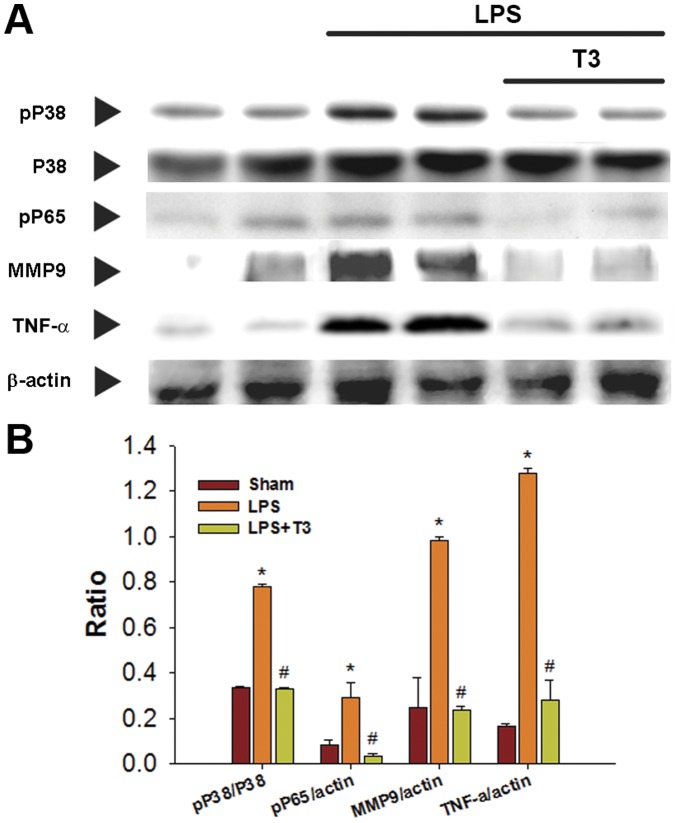
Comparison of the protein expression of phospho-p38, total p38, phospho-p65, MMP9, and TNFα between sham, LPS, and LPS plus 3 mg/kg Thaliporphine (LPS/T3) groups. Representative Western blots are shown at top **A**. and densitometric analyses at bottom **B**. The data of phospho-p65, MMP9, and TNFα were normalized with β–actin and phospho-p38 was normalized with total P38. Plots represent means+S.E. * P<0.05 as compared with sham group; ^#^ P<0.05 as compared with LPS group.

**Figure 3:**
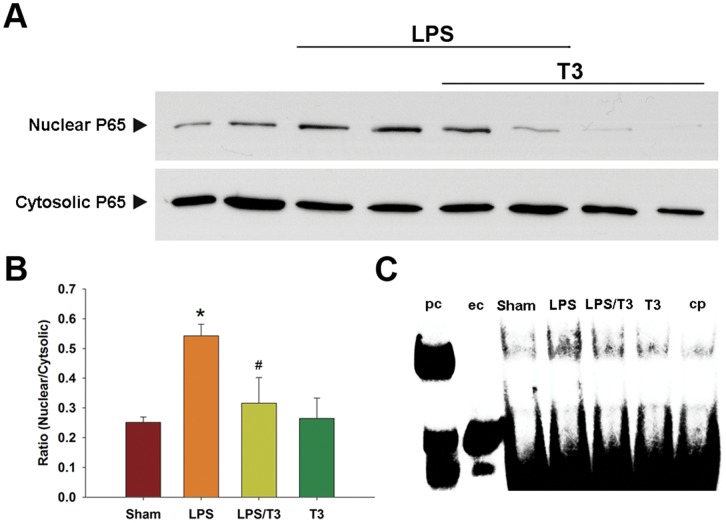
Comparison of the NFκB translocation between sham, LPS, LPS plus 3 mg/kg Thaliporphine (LPS/T3), and Thaliporphine alone (T3) groups. **A**. Nuclear and cytosolic fractions were separately harvested for Western blot analysis of NFκB p65. **B**. The ratio of nuclear fraction of NFκB to cytosolic fraction was quantified. Plots represent means+S.E. * P<0.05 as compared with sham group; ^#^ P<0.05 as compared with LPS group. **C**. The translocation of NFκB p65 was performed with nuclear fractions in different groups by Electrophoretic mobility shift assay (EMSA). The arrows indicate the NFκB specific band and free probe. pc, positive control; ec, EMSA control (without probe); cp, cold (unlabeled) probe against the sample from LPS treated group.

**Figure 4:**
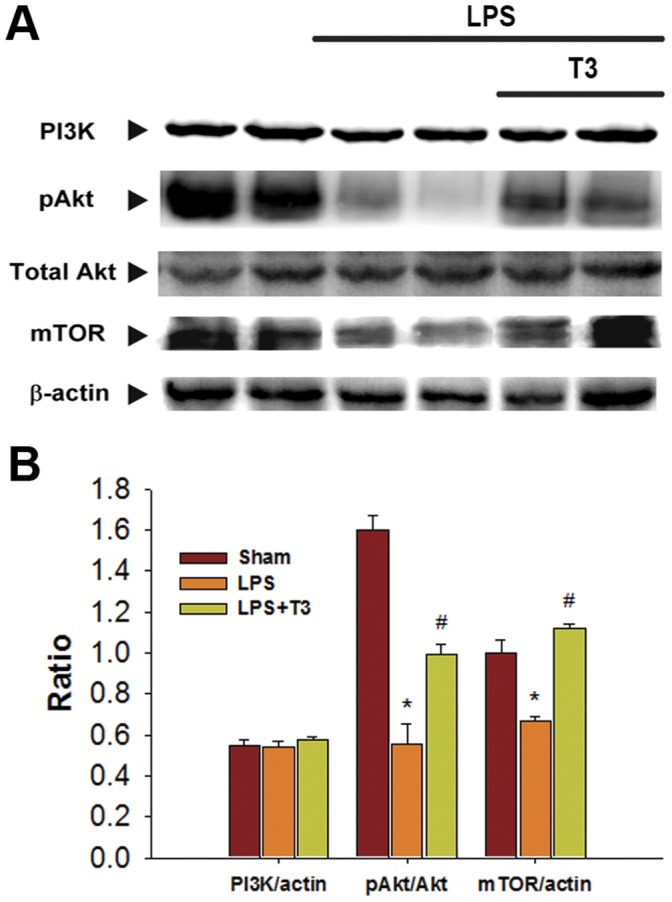
Comparison of the protein expression of PI3K, phospho-Akt, total Akt, and mTOR between sham, LPS, and LPS plus 3 mg/kg Thaliporphine (LPS/T3) groups. Representative Western blots are shown at top **A**. and densitometric analyses at bottom **B**. The data of PI3K and mTOR were normalized with β–actin and phospho-Akt was normalized with total Akt. Plots represent means+S.E. * P<0.05 as compared with sham group; ^#^ P<0.05 as compared with LPS group.

## Results

### 1. Effect of Thaliporphine on Basic and Serum Parameters of Endotoxemic Rabbits

Table 1 summarized and compared some basic and serum parameters between different groups of rabbits. There was no significant difference of mean body weight between four groups, but blood pH and glucose level were both lower after 4 hours LPS injection than were sham (7.45±0.01, 68.17±13.54 mg/dl vs. 7.51±0.02, 157.00±12.73 mg/dl, respectively). Co-administration of Thaliporphine at 1 and 3 mg/kg could dose-dependently recover both blood pH and glucose (7.46±0.04, 114.67±13.29 mg/dl and 7.55±0.02, 143.17±2.95 mg/dl, respectively).

Serum cardiac troponin I (cTnI) and Lactate dehydrogenase (LDH) were also examined. LPS injection increased both cTnI and LDH release (8.70±1.76 ng/ml and 2.2±0.18 compared to sham group 1.08±0.45 ng/ml and 1.31±0.06). Co-administration of Thaliporphine could dose-dependently reduce the cTnI value (6.44±1.74 and 3.98±0.86 ng/ml at dose 1 and 3 mg/kg, respectively), but only 3 mg/kg could significantly decrease the LDH level.

### 2. Effect of Thaliporphine on Hemodynamic Characteristics of Endotoxemic Rabbits

Pressure-volume (P-V) analysis is a useful approach for examining intact chamber function independently of loading conditions. This analysis has been widely used in large animal studies and humans. The systolic and diastolic functions were evaluated *in vivo* at different preloads. After LPS injection (1 mg/kg, *i.v.*) for 4 hours, the basic hemodynamic properties of rabbits were depressed as shown in Figure 1A and summarized in Table 2. There was no significant change in heart rate among four groups. The mean arterial pressures was 78.61±1.68 mmHg in control group, and became 64.48±4.69 mmHg (P<0.02 vs. control) in LPS-group. Co-administration with 1 and 3 mg/kg Thaliporphine could not significantly elevate the MAP of LPS-induced endotoxemic rabbits to control value (60.67±2.20 and 69.93±4.95 mmHg, respectively). Changes detected by conductance catheter readings showed that left ventricular systolic function was impaired after 4 hours in LPS-injected rabbits compared to sham animals. With LPS injection, left ventricular systolic pressure was significantly suppressed to 75.08±4.04 mmHg compared to 96.60±4.92 mmHg of control group. Both +dP/dt and −dP/dt were also significantly lower than in sham-treated animals (2988.08±251.31 vs. 5276.80±777.21 mmHg/s, and 3442.97±514.73 vs. 6413.75±629.40 mmHg/s). Thaliporphine could improve the LVP as the dose increased (75.32±2.17 and 89.95±2.44 mmHg in 1 and 3 mg/kg Thaliporphine groups, respectively), but only high dose (3 mg/kg) Thaliporphine could significantly recover the LPS effects on both +dP/dt and −dP/dt (4075.10±374.31 and 4720.70±400.39 mmHg/s, respectively).

**Figure 5:**
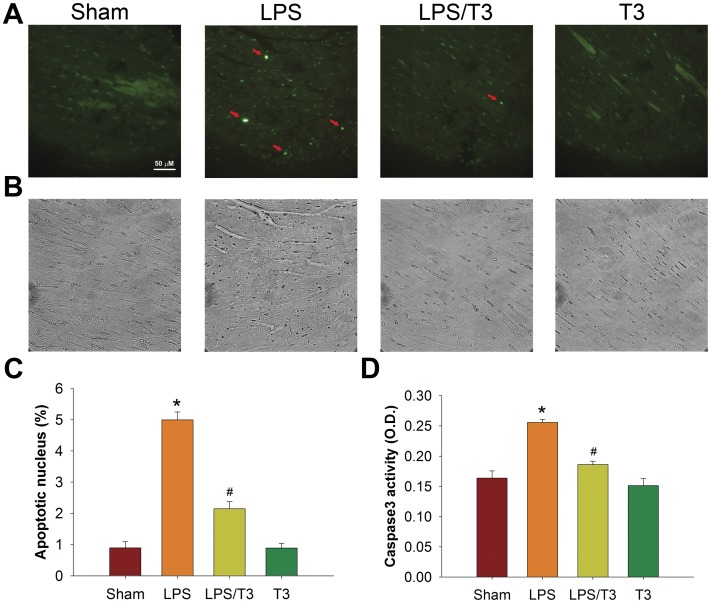
Thaliporphine significantly decreased cardiomyocytes apoptosis. **A**. Apoptosis evidenced by TUNEL staining of apoptotic nuclei in different groups was compared. **B**. The morphology of cells was viewed under a light microscope. **C**. The fraction of apoptotic cells in heart tissue of all groups was quantified by 10 random fields in each slice (four slices in each group). **D**. Caspase3 activity was examined and expressed by means+S.E. * P<0.05 as compared with sham group; ^#^ P<0.05 as compared with LPS group.

Figure 1B displays typical P-V loops obtained after inferior vena cava occlusions in three groups of rabbits. ESPVR was steeper in sham group, suggesting that decreased systolic performance in LPS treated group. On the other hand, the slope of EDPVR was increased in LPS animals, indicating increased end-diastolic stiffness. Co-administration of 3 mg/kg Thaliporphine could reverse both cardiac depressive conditions. In addition to these above parameters, P-V loops at different preloads could be used to derive some other systolic function indexes that were preload-independent, such as Ees, Ea, and PRSW.

Ees is an index of contractility, which is the slope of end-systolic pressure-volume relationship; Ea is a measure of arterial load and is calculated as the simple ratio of ventricular end-systolic pressure to stroke volume; PRSW is the linear regression of stroke work with end diastolic volume. The Ees, Ea, and PRSW, three preload-independent parameters, were all markedly and significantly lower in LPS than in sham groups (49.9±4.8 vs. 85.6±7.8 mmHg/ml, 165.5±30.2 vs. 277.0±29.7 mmHg/ml, 14.13±2.56 vs. 26.97±7.75 mmHg, respectively). Thaliporphine could dose-dependently preserve the Ees and Ea (57.8±8.5 and 227.7±24.3 mmHg/ml at 1 mg/kg, 69.5±5.3 and 238.1±21.6 mmHg/ml at 3 mg/kg), and recover the PRSW (20.90±4.22 mmHg at 1 mg/kg, 27.23±3.81 mmHg at 3 mg/kg).

**Figure 6:**
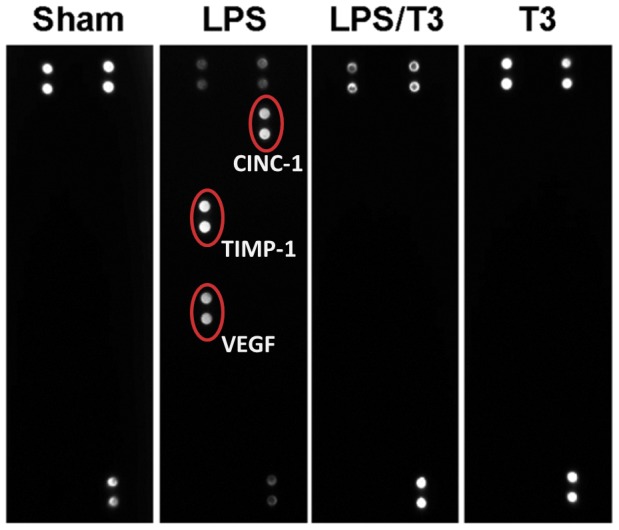
Cytokine protein array analysis of serum from sham, LPS, LPS plus 3 mg/kg Thaliporphine (LPS/T3), and Thaliporphine alone (T3) groups. Red circles indicate differences observed between sham and LPS. CINC-1, TIMP-1, and VEGF were markedly increased in LPS group but not both LPS/T3 and T3 group. The six signal points in the membrane of sham group indicate experimental positive controls.

### 3. Effect of Thaliporphine on p38 MAP Kinase/NFκB and PI3K/Akt/mTOR Signaling in Myocardial Tissues

p38 MAP kinase/NFκB and PI3K/mTOR are two LPS-induced signaling pathways in cardiomyocytes. In 4 hours LPS-treated rabbit hearts, the protein expression levels of phospho-p38 MAP kinase, phospho-p65 NFκB, MMP9, and especially TNFα were all increased as compared with sham (Figure 2). Thaliporphine at 3 mg/kg could diminish these increases significantly without changes of total p38 expression. In LPS group, the abundance of the NFκB p65 subunit was increased in the nuclear fraction and slightly decreased in the cytosolic fraction of rabbit heart tissue, suggesting that LPS induces the translocation of NFκB into the nucleus (Fig. 3A). Thaliporphine at 3 mg/kg significantly reduced the ratio of nuclear fraction of p65 to cytosolic fraction (Fig. 3B). The binding between NFκB p65 and DNA was also enhanced by LPS, and reversed by 3 mg/kg Thaliporphine pretreatment (Fig. 3C). Figure 4 shows that 3 mg/kg Thaliporphine could reverse the decrease of phopho-Akt and mTOR expression by 4 hours LPS treatment. Phospho-Akt was normalized with total Akt expression. However, the expression level of PI3K was not altered with LPS plus Thaliporphine treatment or LPS alone.

### 4. Effect of Thaliporphine on Apoptosis and Caspase3 Activity in Myocardial Tissues

Apoptosis is determined by TUNEL staining and confirmed with caspase3 activity measurement. TUNEL staining enables the highly sensitive detection of apoptosis in tissue and single cells, and Caspase3 is activated in the apoptotic cell both by extrinsic (death ligand) and intrinsic (mitochondrial) pathways. After four hours treatment with 1 mg/kg LPS, the fraction of TUNEL (Fig. 5A) positive nuclei was increased from 0.90±0.20% to 4.99±0.26% (Fig. 5C), which was consistent with the increase of caspase3 activity (Figure 5D). Co-administration of 3 mg/kg Thaliporphine reduced both caspase 3 activity and cell apoptosis.

### 5. Effect of Thaliporphine on Cytokine Release

Serum from LPS injected group showed a marked increase in the expression of three cytokines, including cytokine-induced neutrophil chemoattractant (CINC)-1, tissue inhibitor of metalloproteinase (TIMP)-1, and vascular endothelial growth factor (VEGF). Pretreated with Thaliporphine at 3 mg/kg abolished the expression of these proteins. No difference was observed between the effects of Thaliporphine and sham group on cytokine expression (Fig. 6).

## Discussion

Myocardial dysfunction is an important manifestation in sepsis [9], [15]. Decrease in ventricular systolic function have been found consistently in association with sepsis in humans [16], animals [17], isolated hearts or papillary muscle of animal models [18]. Most experimental models, such as endotoxemic animals, reveal hypovolemia, which is not the direct evidence for myocardial depression. Both elevated sympatho-adrenal activation and decreased vascular resistance in endotoxemia may mask myocardial depression [19]. Coronary circulation, myocardial metabolism, and humoral mediators all play important roles in myocardial dysfunction. Cardiac depression of human sepsis is not caused by the hypotension and hypoperfusion of the shock state since septic patients have high coronary flow and diminished coronary artery-coronary sinus oxygen difference [20]. Discussions on the true involvement of the heart in sepsis and septic shock, regardless of hemodynamic conditions, date back to the early 1960s [21], when some studies already used endotoxic shock models in animals. Although the effects of sepsis on cardiac function have been the subject of much investigation, the pathophysiology of this condition remains incompletely understood [22]. There were mechanisms involved in the pathophysiology of myocardial dysfunction in sepsis include inflammatory, mitochondrial dysfunction, autonomic dysregulation, and cell death [23].

Left ventricular pressure has been widely used as the cardiac contractile parameters, but it is recognized that they are all preload dependent, which cannot reliably be used in models that preload are altered [24]. Therefore, the pressure-volume (P-V) relationship obtained by varying the cardiac loading is widely used, which is relatively specific index for preload-independent contractility [25]. In endotoxemic volunteers or animals, this relationship is decreased, even without hypotension [26], [27]. In the present study, not only preload-dependent parameters LVSP, +dP/dt, and -dP/dt, but also preload-independent indexes of cardiac contractility, Ees (the slope of end-systolic pressure-volume relationship) and PRSW (the linear regression of stroke work with end diastolic volume) were measured and found all these parameters were decreased in LPS group compared to sham group. Our findings of impaired LV systolic function in septic rabbit, especially preload-independent parameters after LPS treatment, are consistent with previous observations by others [28].

Although there are abundant studies regarding to the mechanisms of LPS induced cardiac depression, the main mechanisms remain controversial [9], [15], [29]. LPS activates a broad range of signaling pathways in cardiomyocytes, including MAP kinase and NFκB cascade, which induced complicate inflammatory cascades, cytokine release, and apoptosis [30]. Three types of MAP kinases (ERK1/2, p38, and JNK) can be activated by LPS in cardiomyocytes [31], [32]. They played roles in the activation of NFκB signaling, release of cytokines, and conversion of the myocytes to a proinflammatory phenotype [30]. These findings are in general agreement with previous studies implicating the role of p38 MAP kinase on the production of cytokines (TNFα- and IL-1β) from cardiomyocytes by LPS treatment [33], [34]. NFκB signaling is responsible for not only the generation of these inflammatory factors but also their effects on inflammation and apoptosis [35], [36], [37]. Septic serum can be viewed as a pool of inflammatory factors, such as IL-1β- or TNFα, both from myocytes themself or other cells have potential to activate many signaling pathways of cardiomyocytes. In this study, we found LPS could significantly induce CINC-1, TIMP-1, and VEGF release, and Thaliporphine at 3 mg/kg significantly reverse the effects with cytokine protein array. We also measured IL-1β, IL-6, and IL-10 level with ELISA kits for human sample. However, maybe because of the species difference or sensitivity, we could not get any meaningful data from all samples (data not shown). Accumulating evidences indicate that myocardial TNFα (versus systemically) is an autocrine contributor to myocardial dysfunction and apoptosis in sepsis and chronic heart failure [38]. TNFα was reported to cause cardiomyocytes apoptosis and followed by decrease of myocyte contraction by about 25% [38], [39].

Not only the main signaling pathway LPS induced, recent study suggests LPS also stimulates PI3K/Akt/mTOR pathway and demonstrates mTOR has a strong negative regulatory role in controlling NFκB activation and MMP9 expression in different cell models [11], [40]. mTOR could decrease LPS-induced mitogen- and stress-activated protein kinase-1 (MSK1) activation through reducing phosphorylation of p38 MAP kinase, which acts upstream of NFκB [41]. MMP9 plays a pivotal role in the turnover of extracellular matrix and in the migration of immune cells at injury sites [42], which was also induced by LPS associated NFκB activation.

Thaliporphine, a novel aporphine alkaloid, was proved to increase survival and ameliorate cardiac depression in small endotoxemia animals [13], [14]. In this study, we first demonstrated 3 mg/kg Thaliporphine could restore both preload-dependent and independent hemodynamic cardiac depression parameters of large endotoxemic animal, although 1 mg/kg Thaliporphine could only reverse some of them. Our results indicate Thaliporphine protect rabbit heart from endotoxemic injury by decreasing p38 MAP kinase, NFκB, MMP9, TNFα expression and increasing PI3K/Akt/mTOR pathway. The decrease of “bad” and increase of “good” pathways resulted in following protection effects against cardiomyocytes apoptosis and depression. On the other hand, in our sepsis model we also found the expression of Bcl-2 of cardiac tissues were increased by Thaliporphine, which could counteract LPS induced apoptosis (data not shown).

In our previous study, Thaliporphine could ameliorate LPS-induced cardiac depression through attenuating TLR4 signaling in cardiomyocytes to prevent SERCA2 from nitrosylation via decreasing iNOS and TNFα expression [14]. However, change of SERCA2 nitrosylation was not observed in this study (data not shown). Endotoxic shock has been shown to impair SERCA2 activity in subcellular fractions in some [43], [44] but not all studies [6], [45], [46]. This discrepancy may be explained by inter-study variations such as different cardiac loading, circulating catecholamines, animal models, materials to induce sepsis, or induction periods. That also means Thaliporphine protects heart from cardiac depression by decreasing SERCA2 nitrosylation is not the only mechanism. In other studies, it was also proved that intrinsic myocardial depression is largely related to LPS-mediated sarcolemmal alterations, which shorten action potential duration, and is not due to alterations in SR function [6].

Serum levels of cardiac enzymes such as CKMB and LDH are essential to the diagnosis of myocardial damage. A new serum marker, cardiac troponin I (cTnI), has been developed for the detection of cardiac injury and diagnosis of septic myocarditis [47]. It is specific for cardiac tissue and is detected in the serum only if myocardial has been injured. It is considered to be more sensitive and specific than CKMB and LDH [48]. Polymorphonuclear neutrophil (PMN) infiltration in the myocardial interstitum is also an important event to myocardial dysfunction in sepsis [49], [50]. PMN is the most abundant white blood cells and is an important part of the innate immune system. Animal Studies indicate PMN activation can directly injure cardiomyocytes [51]. In this study, Thaliporphine could reverse the LPS-induced dramatic increases of both cTnI and LDH without significant changes of PMN and LYM. Thus the decreases in myocardial injury markers were not related to changes in circulating PMN. The LPS induced increases in these markers were accompanied by decreases in myocardial contractility as we mentioned above.

The mechanisms involved in myocardial dysfunction in sepsis is multifactorial, inflammatory cytokines, NO, and other factors all play important roles [7], [8], [9]. According to our previous data in endotoxemic rat, the serum level of nitrate was increased up to 10 folds than sham group after 4 hours injection of LPS [13]. However, the administration of L-arginine analogues *in vivo*, competing with natural L-arginine for nitric oxide synthase (NOS), does not ameliorate cardiac depression, suggesting that NO may not play a significant role in heart dysfunction *in vivo* [52]. Intracellular acidosis has negative inotropic effects by impairing excitation-contraction coupling and decreasing myofilament sensitivity to Ca^2+^ [53]. Although serum acidosis may produce intracellular acidosis in hearts, the intact heart appears more resistant to acidosis [54]. The serum acidosis may be rapidly corrected by adjusting the respiratory rate or other compensation mechanisms *in vivo*. Therefore, it is unlikely that transient serum acidosis contributed to the sustained cardiac depression. In the present study, we found serum pH was slightly decreased in LPS treated rabbits, but not to the acidosis degree and this decrease was reversed with Thaliporphine administration. In conclusion, we first found that Thaliporphine could protect sepsis-associated myocardial dysfunction in both preload-dependent and independent ways. This protective effect is related to the increase of “good” – PI3K/Akt/mTOR and decrease of “bad” – p38/NFκB pathways, which is associated with diminishing TNFα and caspase 3 dependent apoptosis.
